# Adjuvant therapeutic potential of tonabersat in the standard treatment of glioblastoma: A preclinical F98 glioblastoma rat model study

**DOI:** 10.1371/journal.pone.0224130

**Published:** 2019-10-21

**Authors:** Valerie De Meulenaere, Ellen Bonte, Jeroen Verhoeven, Jean-Pierre Kalala Okito, Leen Pieters, Anne Vral, Olivier De Wever, Luc Leybaert, Ingeborg Goethals, Christian Vanhove, Benedicte Descamps, Karel Deblaere

**Affiliations:** 1 Department of Radiology, Ghent University Hospital, Ghent, Belgium; 2 Department of Pharmaceutical analysis, Ghent University, Ghent, Belgium; 3 Department of Neurosurgery, Ghent University Hospital, Ghent, Belgium; 4 Department of Human Structure and Repair, Ghent University, Ghent, Belgium; 5 Department of Experimental Cancer Research, Ghent University, Ghent, Belgium; 6 Department of Basic Medical Sciences, Ghent University, Ghent, Belgium; 7 Department of Nuclear Medicine, Ghent University Hospital, Ghent, Belgium; 8 IBiTech—Medisip—Infinity lab, Ghent University, Ghent, Belgium; George Washington University, UNITED STATES

## Abstract

**Purpose:**

Even with an optimal treatment protocol, the median survival of glioblastoma (GB) patients is only 12–15 months. Hence, there is need for novel effective therapies that improve survival outcomes. Recent evidence suggests an important role for connexin (Cx) proteins (especially Cx43) in the microenvironment of malignant glioma. Cx43-mediated gap junctional communication has been observed between tumor cells, between astrocytes and between tumor cells and astrocytes. Therefore, gap junction directed therapy using a pharmacological suppressor or modulator, such as tonabersat, could be a promising target in the treatment of GB. In this preclinical study, we evaluated the possible therapeutic potential of tonabersat in the F98 model.

**Procedures:**

Female Fischer rats were inoculated with ± 25.000 F98 tumor cells in the right frontal lobe. Eight days post-inoculation contrast-enhanced T1-weighted (CE-T1w) magnetic resonance (MR) images were acquired to confirm tumor growth in the brain. After tumor confirmation, rats were randomized into a Control Group, a Connexin Modulation Group (CM), a Standard Medical Treatment Group (ST), and a Standard Medical Treatment with adjuvant Connexin Modulation Group (STCM). To evaluate therapy response, T2-weighted (T2w) and CE-T1w sequences were acquired at several time points. Tumor volume analysis was performed on CE-T1w images and statistical analysis was performed using a linear mixed model.

**Results:**

Significant differences in estimated geometric mean tumor volumes were found between the ST Group and the Control Group and also between the STCM Group and the Control Group. In addition, significant differences in estimated geometric mean tumor volumes between the ST Group and the STCM Group were demonstrated. No significant differences in estimated geometric mean tumor volumes were found between the Control Group and the CM Group.

**Conclusion:**

Our results demonstrate a therapeutic potential of tonabersat for the treatment of GB when used in combination with radiotherapy and temozolomide chemotherapy.

## Introduction

Gliomas form a heterogeneous group of tumors of the central nervous system (CNS) and are categorized into 4 histological grades based on increasing degrees of undifferentation, anaplasia and aggressiveness as described by the World Health Organisation (WHO) classification system [1]. Recently, an updated WHO classification system was released, including histological criteria and molecular biomarkers [2]. Malignant gliomas are by far the most common form of glioma and glioblastoma (GB) accounts for 82% of cases of malignant glioma [3]. Therefore, GB is the most common and malignant glial tumor of the CNS with a global incidence which ranges from 0.59 to 3.69 per 100 000 depending on the reporting country/organization [4]. Up to now, Magnetic Resonance Imaging (MRI) is the gold standard for the clinical evaluation of GB and is well suited for longitudinal follow-up. Gadolinium (Gd)-based contrast-enhanced (CE) MRI is widely considered as the most accurate imaging tool for diagnosis of malignant glioma [3,5]. Standard medical treatment of GB consists of maximal surgical resection followed by radiotherapy (RT) and concomitant chemotherapy with temozolomide (TMZ) [6]. Unfortunately, despite this multimodal treatment method, standard medical treatment has limited efficacy due to high rates of recurrence, overall resistance to therapy, and neurological deterioration [7]. Therefore, GB is associated with poor prognosis as the median patient survival is 12.1–14.6 months from diagnosis, and only 3–5% of the patients survive for three years or longer [6]. Hence, there is an urgent need for novel effective therapies that act on GB and improve survival outcomes.

Recent evidence suggests an important role for transmembrane connexin (Cx) proteins in the microenvironment of malignant glioma [8]. Cxs are proteins that form hemichannels (HCs) when six of the same Cxs are grouped [9]. The docking of two HCs, belonging to the membrane of neighboring cells, results in the formation of gap junctional channels (GJs), i.e., intercellular channels that allow the direct exchange of ions and low molecular weight molecules (<1.5kDa) between the cytoplasm of neighboring cells [9]. GB cells can interconnect via microtube-associated gap junctions (tumor microtubes: TMs) based on Cx43. These TMs allow gap junction-mediated communication (GJC) between tumor cells [10]. Cx43 is suggested to be a driver of tumor invasion, a marker of tumor progression and an inducer of TMZ resistance in GB cells [8]. TMs also play an important role in potential resistance against radiotherapeutic damage. Radiotherapy causes increases of intracellular calcium levels, which are necessary for radiotherapy-induced cytotoxicity. Intercellular TMs can distribute these critical levels of calcium within the larger cellular network causing nonlethal calcium levels [10]. Moreover, GJC has also been described between glioma cells and astrocytes and is critical for several physiological processes. Astrocytes are the most dominant cell type in the brain and maintain homeostasis of the brain microenvironment [11]. In normal physiological conditions, the network of astrocytes has a protective role in the CNS but, under pathological conditions, astrocytes become activated to protect neurons from injury-induced apoptosis via GJC [11]. Therefore, gap junction directed therapy using a pharmacological suppressor or modulator of gap junction activity such as tonabersat could be a promising target in the treatment of GB. Tonabersat is a benzopyran derivate that binds to a unique stereoselective binding site in astrocytes and inhibits gap junction mediated processes [12]. Moreover, it has been shown that tonabersat directly reduces opening of HCs under pathological conditions [13]. Tonabersat was also thought to inhibit cortical spreading depression, which is a key mechanism underlying the depolarizing brain waves in migraine with aura. Based on preclinical results, tonabersat was selected for phase II clinical trials as a prophylactic treatment for migraine, and it was shown to have a preventive effect on attacks of migraine with aura [14].

Currently, several brain tumor models are used for GB research. We selected the F98 GB model as it displays all the necessary histological characteristics, is non-immunogenic and less expensive compared to patient-derived GB models necessitating the use of nude rats [15].

In this preclinical study, we evaluated the possible adjuvant therapeutic effect of tonabersat in the F98 GB rat model.

## Materials and methods

### Cell culture

The F98 cell line (ATCC® CRL-2397^™^) was generated from Fischer rats after treatment with N-ethyl-N-nitrosourea and has previously been described in detail [16]. F98 GB cancer cells were maintained in Dulbecco’s modified Eagle’s medium (DMEM) supplemented with 10% fetal calf serum (FCS), 1% penicillin-streptomycin antibiotics, 0.0005% fungizone and 1% pyruvate at 37°C and 10% CO_2_.

### F98 GB rat model

This study protocol was approved by the Ghent University ethics committee for animal experiments (ECD 17/70). All animals were kept and handled according to the European guidelines (directive 2010/63/EU) and housed under environmentally controlled conditions (12h normal light/dark cycles, 20°C– 24°C and 40–70% relative humidity, daily monitoring) with food and water ad libitum.

The F98 GB rat model has previously been described by Bolcaen et al. [17]. Female Fischer rats (F344/IsoCrl, Charles River®, body weight 174 ± 10 g, mean ± SD) were anesthetized with an intraperitoneal (IP) injection of a mixture of 74 mg/kg ketamine and 11 mg/kg xylazine and immobilized using a stereotactic frame (Model 902 Dual Small Animal Stereotaxic frame, Kopf®). Subsequently, the rat head was shaved, swabbed with chlorhexidine and a longitudinal scalp incision of 10 mm was made. Next, a hole of 1 mm was made through the skull in the right frontal hemisphere (2 mm posterior and 2.5 mm lateral to bregma) with the use of a diamond drill (Dremel®). Then, a stereotactically guided syringe, secured into a holder, containing a 5 μl cell suspension of 25 000 F98 cancer cells was slowly inserted at a depth of 3 mm and the cell suspension was injected over a 2-min period using a microsyringe pumpcontroller (Micro 4TM, World Precision Instruments, Sarasota, USA). The syringe was withdrawn 5 minutes post inoculation (PI) and the incision was closed with bone wax (Aesculap AG®) and sutured.

Taking the humane endpoints into account, animals were immediately euthanized when clinical or behavioral signs such as balance problems, deterioration, weakness, reduced activity, absence of grooming or weight loss (> 20%) were observed. Moreover, tumor growth was monitored by brain MRI (euthanasia when tumor volume > 40% of total brain volume). An overview of day and cause of euthanasia for all rats can be found in the supporting information (S1 Table). All animals were euthanized based on the humane endpoints.

### MRI for confirmation of GB growth

Eight days PI, MRI was performed on a 7T system (PharmaScan 70/16, Bruker, Ettlingen, Germany) to confirm GB growth in the rat brain. Rats were anesthetized using isoflurane mixed with oxygen administered at a flow rate of 0.2 l/min (induction 5%, maintenance 1.5%). Then, a Gd-based contrast agent (2 mmol/kg, Dotarem, Guerbet) was intravenously (IV) injected into a tail vein followed by fixation of the rat through the nose cone on the restrainer. A heating pad was placed beneath each rat to maintain body temperature at 37°C before they were placed inside the magnet. A rat brain surface coil (Rapid Biomedical, Rimpar®, Germany) was applied around the head followed by positioning of the bed in a 72 mm whole body transmitter coil (Rapid Biomedical, Rimpar®, Germany). T2-weighted (T2w) sequences (SE TurboRARE, 109 μm in-plane resolution, TR/TE 3661/37.1 ms, 4 averages, TA 9’45") were performed to localize the tumor and to assess tumor growth. Contrast-enhanced T1-weighted (CE-T1w) sequences (SE RARE, 117 μm in-plane resolution, TR/TE 1539/9.7 ms, 3 averages, TA 4’15”) were acquired to demonstrate the increased permeability of the blood brain barrier (BBB) present in GB and to measure tumor volumes.

### Treatment groups

**Fig 1:**
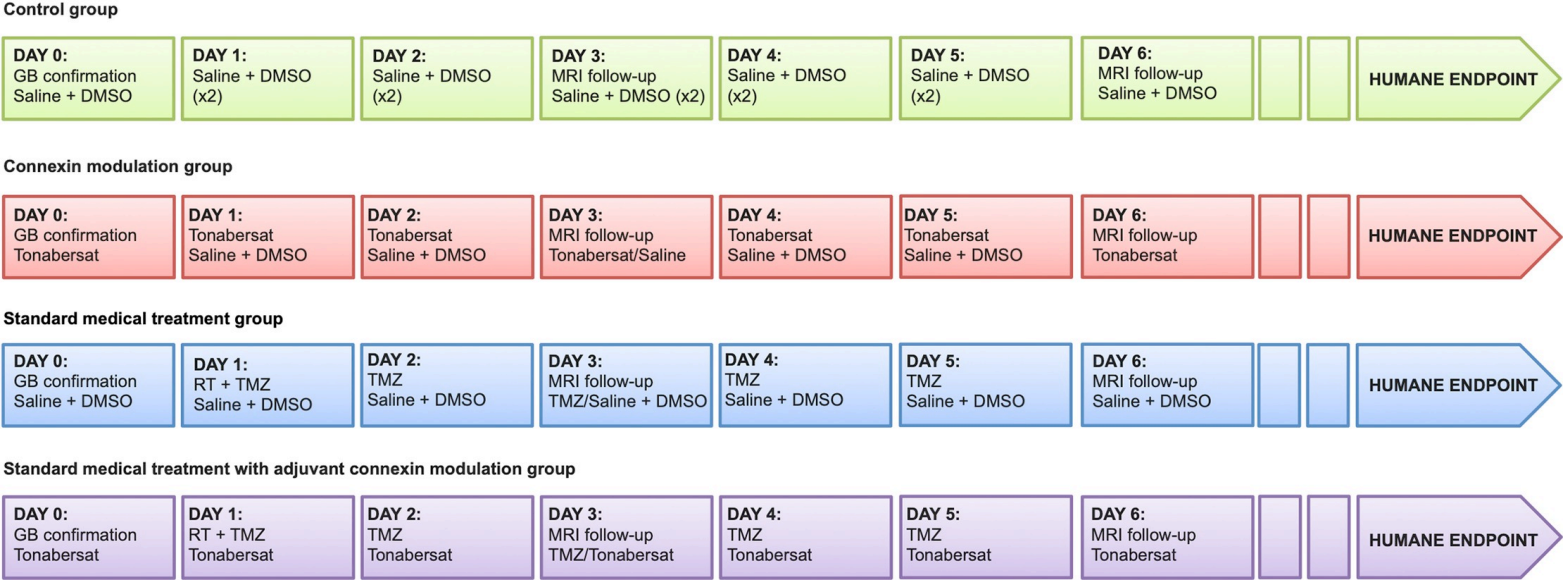
Overview of the protocol per group.

When the diameter of the tumor was approximately 2–3 mm on the CE-T1w MR image, the rats were randomized into a Control Group (n = 10), a Standard Medical Treatment Group (ST, n = 6), a Connexin Modulation Group (CM, n = 5), a Standard Medical Treatment and Connexin Modulation Group (STCM, n = 8) (Fig 1).

### Control Group

The rats in the Control Group received daily IP injections of saline with 20% dimethylsulfoxide (DMSO, Sigma-Aldrich). These injections were performed to take into account a possible influence of DMSO that was used to dissolve TMZ and tonabersat. The control injections were performed until the humane endpoints were reached.

### CM Group

Therapy with tonabersat as a single agent (10 mg/kg; MedChem Express) was performed through daily IP injections starting at tumor confirmation until humane endpoints were reached. Tonabersat was dissolved in 10% DMSO and diluted with phosphate-buffered saline (PBS) to max 1 ml.

### ST Group

The rats in the ST Group received MR-based RT with concomitant chemotherapy with TMZ (IP injections for 5 days; 29 mg/kg).

MR-based RT. Radiotherapy treatment was performed using MRI-guided conformal arc treatment with the Small Animal Radiation Research Platform (SARRP, XStrahl, UK). After IV injection of a Gd-based contrast agent, the rat was fixed on a multimodality bed. Two water filled capillaries were placed on the rat head and were used as markers to facilitate co-registration of the CT scan and the MRI scan. CE-T1w sequences were run and the rat was, fixed on the multimodality bed, transported to the four-axis robotic positioning stage of the SARRP. First, a treatment planning CT (360 projection acquired over 360 degrees using a 1 mm aluminium filter, 1 min acquisition) with 600 μA tube current and 50 kV tube voltage was performed. Then, the CT data were reconstructed using an isotropic voxel size of 0.2 mm and imported in the treatment planning software (Muriplan, Version 2.0.6, Xstrahl®, UK) to distinguish air, soft tissue and bone by manual segmentation based on the grey-value histogram. Co-registration with the MRI was manually done using the capillary markers and the skull. Based on the CE-T1w MR scan the isocenter of the radiation bundle was set in the middle of the tumor region. A single dose of 20 Gy was delivered by applying three non-coplanar arc beams (120°) using a 5 x 5 mm collimator to include minor position changes of the rat head during performance of the treatment (voltage X-ray source: 220kV, tube current: 13 mA and copper filter of 0.15 mm) [18].

Chemotherapy with temozolomide. TMZ (29 mg/kg; MedChem Express) was dissolved in 20% DMSO and diluted with saline to max 1 ml. IP injections of TMZ were performed for 5 consecutive days starting at the day of RT.

### STCM Group

In addition to the standard medical treatment, rats from the STCM received connexin modulation with tonabersat (10 mg/kg; MedChem Express). Tonabersat was dissolved in 10% DMSO and diluted with phosphate-buffered saline (PBS) to max 1 ml. Tonabersat were administered through daily IP injections, starting from tumor confirmation (i.e. one day before RT and chemotherapy with TMZ) until humane endpoints were reached. To avoid a possible interaction between IP injection of tonabersat and IP injection of TMZ, the injections were given with minimum 8 hours difference, which was an arbitrary choice.

### MRI for follow-up of GB growth

To evaluate therapy response, T2w and CE-T1w MRI sequences were run at day 3, 6, 10, 13, then every two days until day 39 (day 1 = start of standard medical treatment or first control injection).

### Image analysis

Tumor volumes were measured on CE-T1w MR images by manually outlining the hyperintense regions on individual slices using OsiriX software (OsiriX v.5.8.1). The obtained tumor areas were then multiplied by the slice thickness (0.6 mm) to calculate GB volume.

### Statistical analysis

Traditional ANOVA and regression models assume that individual observations are uncorrelated. In the present study, rats were randomized into treatment groups and multiple tumor volume measurements per rat were performed at several time points. Rats were euthanized at different time points based on their clinical symptoms resulting in missing data that meet the missing-at-random principle (i.e., missing data are dependent on therapy and pretreatment tumor volume, but independent on unobserved variables). Moreover, the clustered structure of the data (i.e., several series of rats were inoculated with cancer cells) required a multilevel approach. Mixed model analysis provides a general, flexible approach in these situations and is therefore the most suitable statistical model.

A linear mixed model was fitted for the natural logarithm of tumor volume, with treatment group, time since tumor confirmation (in days, categorical), their two-way interaction (treatment group * time categorical) and the natural logarithm of pretreatment tumor volume (in mm^3^, continuous) in the fixed part of the model, and with a random intercept for series and a first order autoregressive correlation structure for repeated measurements within the same animal. Results were backtransformed by taking the exponential of the regression coefficients. The exponentiated regression coefficients correspond to changes in the ratio of the expected geometric mean tumor volume.

Results were visualized in a mean profile plot showing the estimated geometric mean tumor volume according to the treatment group they belong to, and as a function of time since tumor confirmation in days. Estimates are derived from a model adjusted for pretreatment tumor volume and correspond to the geometric mean tumor volume when the pretreatment volume is 17.7 mm^3^ (17.7 mm^3^ is the geometric mean tumor volume at tumor confirmation and is a criterion for the central tendency). A similar model was fitted on all data available of the first two weeks since tumor confirmation, with time since tumor confirmation (in days) as a continuous covariate instead of categorical, to estimate the percentage increase in the expected geometric mean tumor volume per day during the first two weeks for the different treatment groups. The slopes were compared with those of the control group.

Unadjusted p-values for two-sided tests and 95% confidence intervals are reported. However, when applying Bonferroni correction, p-values should be compared to a significance level of 0.13% because in total 38 comparisons were made. Analyses were performed in SPSS, version 25. Figures were made in R, version 3.5.2.

### Histological analysis

When humane endpoints were reached, animals were euthanized by an IV injection of pentobarbital (120 mg/kg). For a selection of animals the brains were isolated, immersed for 24 hours in 4% paraformaldehyde and embedded in paraffin. Then, the cerebrum was partly sectioned in 5μm slices and stained with Hematoxylin and Eosin (H&E) for histological confirmation of GB development.

Immunohistochemical staining for Ki67, Cx43 or Glial Fibrillary Acidic Protein (GFAP) were performed on formalin-fixed, paraffin-embedded slices to evaluate GB proliferation, Cx43 expression and reactive astrocytes, respectively. Sections were incubated for 30 min with normal swine serum (Ki67 and Cx43) or normal rabbit serum (GFAP), followed by incubation with the primary antibodies: rabbit monoclonal (Ki67: 1/50, 2h, Invitrogen MA5-14520); Cx43: 1/2000, 2h, Abcam ab11370) or mouse monoclonal (GFAP, 1/400, overnight, ThermoFisher, MA5-12023). Then, sections were incubated with biotinylated secondary antibodies (1/200, 30 min), streptavidin-peroxidase complex (1/200, 30 min) and 3,3′- diaminobenzidine (DAB) peroxidase solution (10 min). Finally, sections were counterstained with haematoxylin (Mayer) and coverslipped using mounting medium (4111, Richard-Allan Scientific, Thermo Fisher Scientific) and glass coverslips (24 × 50 mm #1 (631–0146, VWR)).

All sections were digitally scanned at high resolution (40× magnification) with a virtual scanning microscope (Olympus BX51, Olympus Belgium SA/NV, Berchem, Belgium).

## Results

### Follow-up of GB growth: Mean profile plot

**Fig 2:**
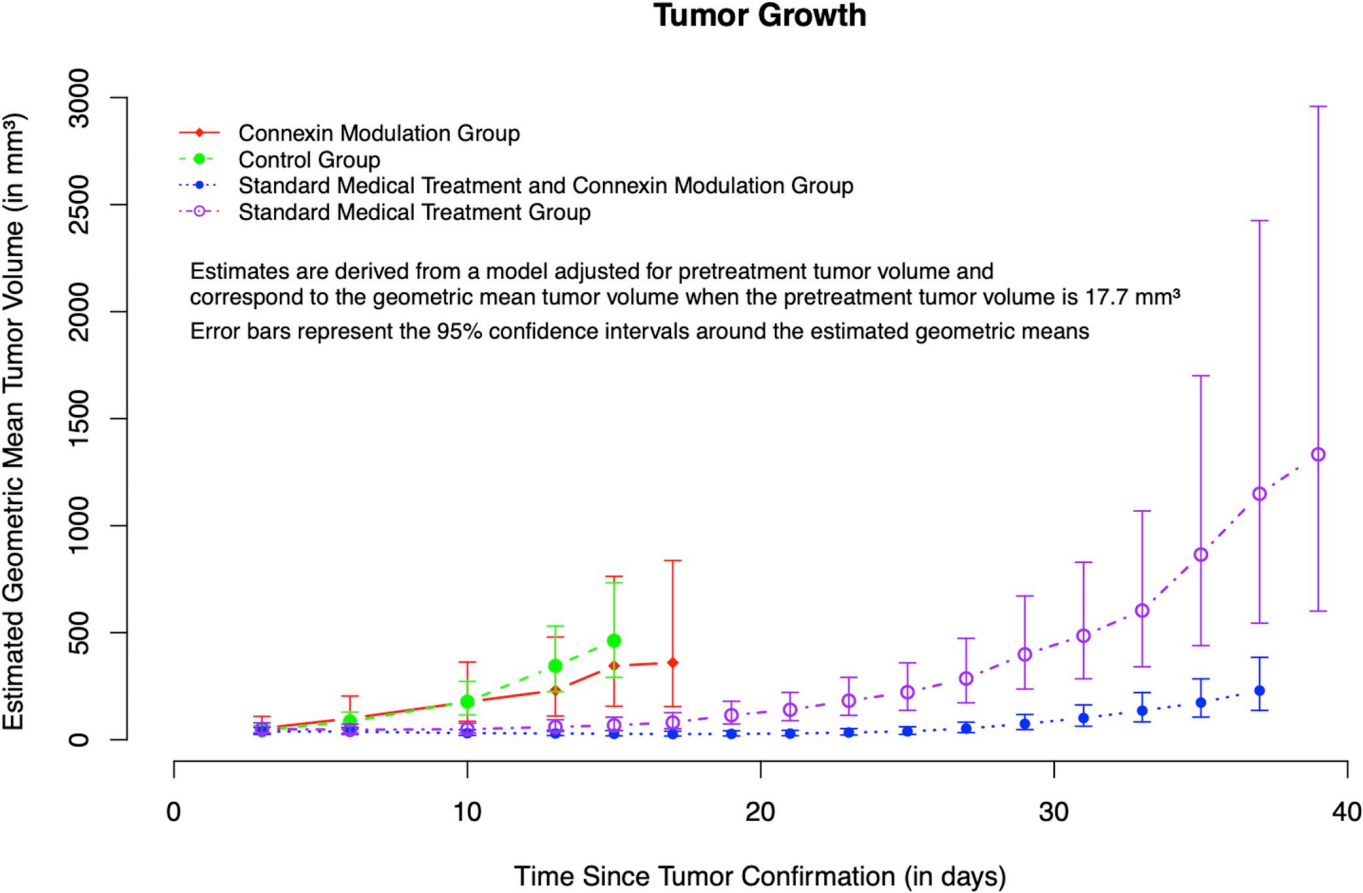
Graphical visualization of tumor growth per treatment group. Estimated geometric mean tumor volumes according to treatment group as a function of time since tumor confirmation in days for animals with a geometric mean pretreatment tumor volume of 17.7 mm^3^.

T2w and CE-T1w MR images were acquired for follow-up of GB growth. Eight days after inoculation of F98 cancer cells, the first hyperintensities due to GB growth were visible on T2w and CE-T1w images. CE-T1w images were used for analysis of GB volumes. Fig 2 and Table 1 show the estimated geometric mean tumor volumes according to treatment group as a function of time since tumor confirmation in days for animals with a pretreatment tumor volume of 17.7 mm^3^ (i.e., the geometric mean pretreatment tumor volume). The measured tumor volumes can be found in the supporting information (S2 Table).

**Table 1:** Overview of estimated geometric mean tumor volumes.

Time since Tumor Confirmation (in days)	Control Group	Connexin Modulation Group	Standard Medical Treatment Group	Standard Medical Treatment and Connexin Modulation Group
3	43.8 (28.7–66.8)	52.7 (25.6–108.3)	50.3 (32.2–78.6)	38.1 (25.1–57.6)
6	84.5 (55.4–129)	99.3 (48.3–204)	46.4 (29.7–72.6)	37.7 (24.9–57.1)
10	177.7 (115.9–272.3)	176.4 (85.8–362.5)	49 (31.4–76.7)	30.4 (20.1–46.1)
13	344.6 (223.7–530.9)	230.5 (110.7–479.7)	59.6 (38.1–93.2)	29.1 (19.1–44.3)
15	462.2 (291.5–733.1)	345.2 (156.2–762.9)	67.4 (43.1–105.4)	27.6 (18.1–42)
17		359.8 (154.7–836.6)	80.5 (51.5–125.9)	26.1 (17.1–39.9)
19			114.6 (73.3–179.2)	27.1 (17.7–41.4)
21			140.1 (88.9–220.8)	28.5 (18.6–43.8)
23			182.1 (113.9–291.3)	33.7 (21.9–51.8)
25			222 (137.2–359.1)	38.9 (25–60.5)
27			285.8 (172.6–473.2)	52.1 (33.2–81.7)
29			398.5 (236.5–671.5)	74.2 (46.9–117.4)
31			485.7 (284.6–829)	101.2 (63–162.5)
33			603.5 (340.7–1068.7)	135.5 (83.4–220.2)
35			864.9 (439.8–1700.8)	173.4 (105.8–284.4)
37			1149 (544.3–2425.6)	229.5 (137–384.3)
39			1333.2 (600.7–2959)	

Estimated geometric mean tumor volumes according to treatment for several time points since tumor confirmation for animals with a geometric mean pretreatment tumor volume of 17.7 mm^3^.

**Fig 3:**
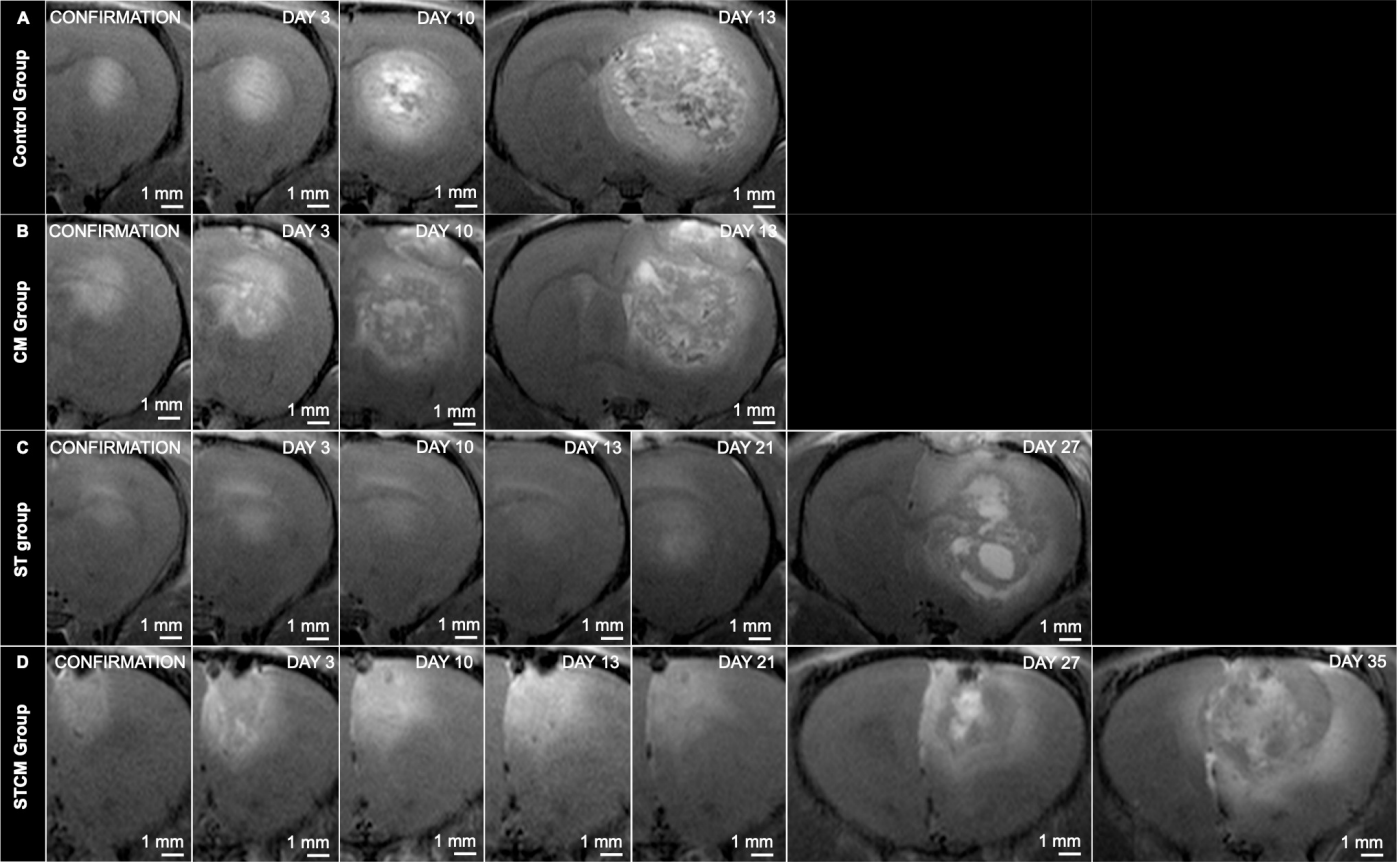
MR imaging of GB growth per treatment group. In vivo serial CE-T1w MRI scans in the same rat showing GB growth in the four treatment groups.

GB in the Control Group showed rapid proliferation encompassing almost the entire right cerebral hemisphere at day 13 to 15, leading to euthanasia of all rats (Fig 3A). At day 13, the estimated geometric mean tumor volume for rats in the Control Group, with a pretreatment tumor volume of 17.7 mm^3^, was 344.6 mm^3^ (95% CI from 223.7 mm^3^ to 530.9 mm^3^). At day 15, the estimated geometric mean tumor volume for rats in the Control Group, with a pretreatment tumor volume of 17.7 mm^3^, was 462.2 mm^3^ (95% CI from 291.5 mm^3^ to 733.1mm^3^) (Table 1).

A similar trend of rapid GB growth was observed in the CM Group. At day 15 to 17, nearly the entire right cerebral hemisphere was covered by GB leading to euthanasia of all animals (Fig 3B). At day 15, the estimated geometric mean tumor volume for rats in the CM Group, with a pretreatment tumor volume of 17.7 mm^3^, was 345.2 mm^3^ (95% CI from 156.2 mm^3^ to 762.9 mm^3^). At day 17, the estimated geometric mean tumor volume for rats in the CM Group with a pretreatment tumor volume of 17.7 mm^3^, was 359.8 mm^3^ (95% CI from 154.7 mm^3^ to 836.6 mm^3^) (Table 1).

GB growth was a slower process in the ST Group and the STCM Group (Fig 3C and 3D). For the ST Group, estimated geometric mean tumor volumes comparable with the end-point estimated geometric mean tumor volumes of the Control Group and the CM Group were reached at day 29 and day 31. At day 29, the estimated geometric mean tumor volume for rats in the ST Group with a pretreatment tumor volume of 17.7 mm^3^, was 398.5 mm^3^ (95% CI from 236.5 mm^3^ to 671.5 mm^3^). At day 31, the estimated geometric mean tumor volume for rats in the ST Group with a pretreatment tumor volume of 17.7 mm^3^, was 485.7 mm^3^ (95% CI from 284.6 mm^3^ to 829 mm^3^) (Table 1). For rats in the STCM Group with a pretreatment tumor volume of 17.7 mm^3^, the maximum estimated mean tumor volume reached was only 229.5 mm^3^ (95% CI from 137 mm^3^ to 384.3 mm^3^) at day 37 (Table 1).

### Evaluation of treatment efficiency via GB volume measurement

Treatment efficiency was evaluated by pairwise comparison of estimated geometric mean tumor volumes at certain time points, assuming they had the same pretreatment tumor volume (Table 2).

**Table 2:** Comparison of estimated mean tumor volumes between treatment groups.

Group	Reference group	Time (days)	Estimated % diff	LCL	UCL	P
Standard Medical Treatment Group	Control Group	3	1.15	0.68	1.94	0.5929
Standard Medical Treatment and Connexin Modulation Group	Control Group	3	0.87	0.53	1.42	0.5643
Standard Medical Treatment and Connexin Modulation Group	Standard Medical Treatment Group	3	0.76	0.47	1.22	0.2479
Connexin Modulation Group	Control Group	3	1.2	0.52	2.76	0.6069
Standard Medical Treatment Group	Control Group	6	0.55	0.33	0.93	0.0263
Standard Medical Treatment and Connexin Modulation Group	Control Group	6	0.45	0.27	0.73	0.0022
Standard Medical Treatment and Connexin Modulation Group	Standard Medical Treatment Group	6	0.81	0.5	1.31	0.387
Connexin Modulation Group	Control Group	6	1.17	0.51	2.7	0.6548
Standard Medical Treatment Group	Control Group	10	0.28	0.16	0.47	< 0.0001
Standard Medical Treatment and Connexin Modulation Group	Control Group	10	0.17	0.1	0.28	< 0.0001
Standard Medical Treatment and Connexin Modulation Group	Standard Medical Treatment Group	10	0.62	0.38	1	0.0516
Connexin Modulation Group	Control Group	10	0.99	0.43	2.28	0.9832
Standard Medical Treatment Group	Control Group	13	0.17	0.1	0.3	< 0.0001
Standard Medical Treatment and Connexin Modulation Group	Control Group	13	0.08	0.05	0.14	< 0.0001
Standard Medical Treatment and Connexin Modulation Group	Standard Medical Treatment Group	13	0.49	0.3	0.79	0.0047
Connexin Modulation Group	Control Group	13	0.67	0.29	1.56	0.3069
Standard Medical Treatment Group	Control Group	15	0.15	0.08	0.26	< 0.0001
Standard Medical Treatment and Connexin Modulation Group	Control Group	15	0.06	0.03	0.1	< 0.0001
Standard Medical Treatment and Connexin Modulation Group	Standard Medical Treatment Group	15	0.41	0.25	0.66	0.0006
Connexin Modulation Group	Control Group	15	0.75	0.3	1.86	0.5049
Standard Medical Treatment and Connexin Modulation Group	Standard Medical Treatment Group	17	0.32	0.2	0.53	< 0.0001
Standard Medical Treatment and Connexin Modulation Group	Standard Medical Treatment Group	19	0.24	0.14	0.39	< 0.0001
Standard Medical Treatment and Connexin Modulation Group	Standard Medical Treatment Group	21	0.2	0.12	0.34	< 0.0001
Standard Medical Treatment and Connexin Modulation Group	Standard Medical Treatment Group	23	0.18	0.11	0.31	< 0.0001
Standard Medical Treatment and Connexin Modulation Group	Standard Medical Treatment Group	25	0.18	0.1	0.3	< 0.0001
Standard Medical Treatment and Connexin Modulation Group	Standard Medical Treatment Group	27	0.18	0.1	0.32	< 0.0001
Standard Medical Treatment and Connexin Modulation Group	Standard Medical Treatment Group	29	0.19	0.1	0.33	< 0.0001
Standard Medical Treatment and Connexin Modulation Group	Standard Medical Treatment Group	31	0.21	0.11	0.38	< 0.0001
Standard Medical Treatment and Connexin Modulation Group	Standard Medical Treatment Group	33	0.22	0.12	0.43	< 0.0001
Standard Medical Treatment and Connexin Modulation Group	Standard Medical Treatment Group	35	0.2	0.09	0.43	0.0001
Standard Medical Treatment and Connexin Modulation Group	Standard Medical Treatment Group	37	0.2	0.09	0.46	0.0002

Pairwise comparison of estimated geometric mean tumor volumes between treatment groups at certain time points, given they had the same pretreatment tumor volume.

At day 3 and 6, no significant differences in estimated geometric mean tumor volumes were observed between the ST Group and the Control Group (p = 0.5929 and p = 0.0263, respectively), between the STCM Group and the Control Group (p = 0.5643 and p = 0.0022, respectively) and between the CM Group and the Control Group (p = 0.6069 and p = 0.6548, respectively).

At day 10, 13 and 15, significant differences in estimated geometric mean tumor volumes were found between the ST Group and the Control Group, indicating that standard medical treatment with radiotherapy and TMZ chemotherapy significantly reduced GB growth from day 10 since tumor confirmation (all p-value < 0.0001). Standard medical treatment supplemented with tonabersat also showed significant differences in estimated geometric mean tumor volumes at day 10, 13 and 15 compared to the Control Group (all p-value < 0.0001). In contrast, no significant differences in estimated geometric mean tumor volumes were found between the Control Group and the CM Group at any time point, indicating that tonabersat as a single agent did not significantly reduce GB proliferation.

Pairwise comparisons of estimated geometric mean tumor volumes were also performed between the ST Group and the STCM Group to evaluate the possible adjuvant effect of tonabersat on standard medical treatment of GB. Statistical analysis showed significant differences in estimated geometric mean tumor volumes between the ST Group and the STCM Group from day 15 onwards (p-value at day 15 = 0.0006, p-values at day 17, 19, 21, 23, 25, 27, 29, 31 and 33 < 0.0001, p-value at day 35 = 0.0001 and p-value at day 37 = 0.0002).

The estimated difference between the estimated geometric mean tumor volume of the ST Group and the STCM Group, assuming that the rats had the same pretreatment tumor volume, was also calculated (Table 2). At day 15, the estimated geometric mean tumor volume of the STCM Group was 0.41 times the estimated geometric mean tumor volume of the ST Group, meaning the estimated geometric mean tumor volume decreases with 59% in the STCM Group compared to the ST Group (95% CI for this ratio from 0.25 to 0.66, p = 0.0006). At day 21, the estimated geometric mean tumor volume of the STCM Group was 0.2 times the estimated geometric mean tumor volume of the ST Group, indicating that the estimated geometric mean tumor volume of the STCM Group is now only 20% of the estimated geometric mean tumor volume of the ST Group (95% CI for this ratio from 0.12 to 0.34, p < 0.0001). From day 21 onwards, the estimated geometric mean tumor volume in the STCM Group was about 20% of the estimated geometric mean tumor volume ST Group at each time point.

### Analysis of daily change in estimated geometric mean tumor volumes between day 3 and 13: Pairwise comparison of the percentage tumor growth

The daily change in estimated geometric mean tumor volume between day 3 and 13 was calculated for all groups (Table 3). For the Control group, the estimated geometric mean tumor volume increases significantly by 22.7% every day (p < 0.001), while in the ST Group, the estimated geometric mean tumor volume only increases with 1.7% (p = 0.321). The difference in daily change between the Control group and the ST Group is highly significant (p < 0.001). Analysis of the daily change in estimated geometric mean tumor volume in the STCM Group shows a decrease of 2.7% every day (p = 0.064), which is also significantly different compared to the daily increase of 22.7% in the Control Group (p < 0.001). These results indicate a slower proliferation of GB in the ST Group and the STCM Group compared to the Control Group. In the Connexin Modulation Group, the estimated geometric mean tumor volume increases significantly with 16.9% every day (p < 0.001), which is not significantly different compared to the Control Group (p = 0.054).

**Table 3:** Pairwise comparisons of the percentage tumor growth between day 3 and 13 for all treatment groups.

Group	% increase per day	LCL	UCL	P	% diff compared to Control	LCL	UCL	P
Control Group	1.227	1.193	1.262	< 0.001				
Standard Medical Treatment Group	1.017	0.984	1.051	0.321	0.828	0.793	0.865	< 0.001
Standard Medical Treatment and Connexin Modulation Group	0.973	0.945	1.002	0.064	0.793	0.761	0.825	< 0.001
Connexin Modulation Group	1.169	1.122	1.218	< 0.001	0.952	0.906	1.001	0.054

### Histological analysis

**Fig 4:**
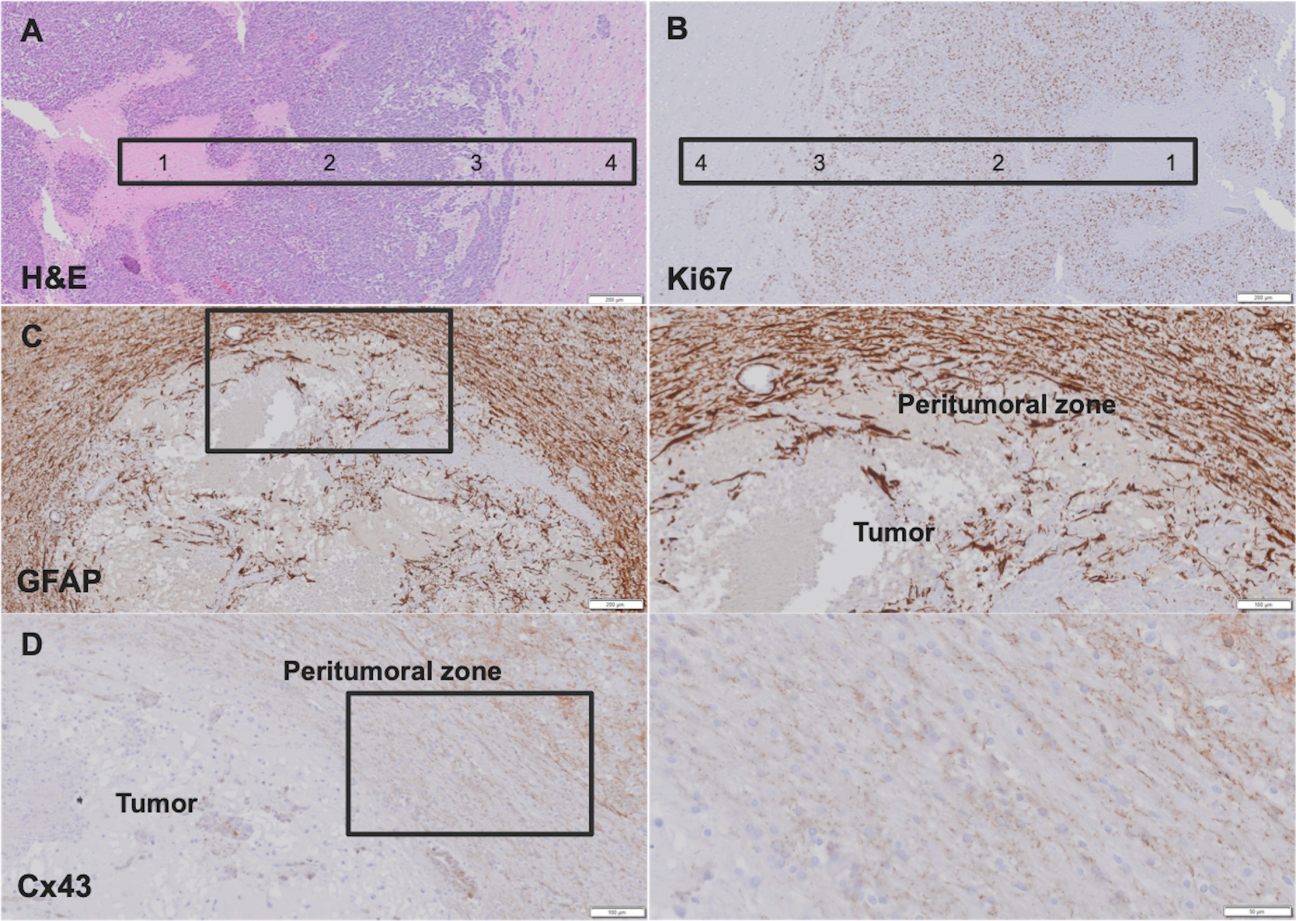
Histological analysis performed on paraffin-embedded slices of resected GB of a rat. A. H&E staining confirmed the presence of GB characterized by central tumor necrosis (1), tumor (2) and the peritumoral zone with infiltrating cancer cells (3) surrounded by healthy brain tissue (4). B. Immunohistochemistry for Ki67 indicated that GB is highly proliferative, except for the necrotic tumor core: tumor necrosis (1), tumor (2), peritumoral zone with infiltrating cancer cells (3) and healthy brain tissue (4). C-D. Immunohistochemistry for GFAP and Cx43 demonstrated GFAP-positive reactive astrocytes and enhanced Cx43 expression at the peritumoral zone.

H&E staining performed on paraffin-embedded slices of a resected GB of a rat at the end of the experiment confirmed GB development in the rat brain (Fig 4A). GB is typically characterized by tumor necrosis (region 1), tumor (region 2) with strong infiltration (region 3) in the surrounding healthy brain tissue (region 4). Ki67 expression indicated that GB was highly proliferative, except for the necrotic tumor core (region 1) (Fig 4B). GFAP-positive reactive astrocytes and enhanced Cx43 expression were present at the peritumoral zone with infiltrating cancer cells, but absent in the necrotic tumor core (Fig 4C and 4D). Cx43 expression is specifically associated with reactive astrocytes (GFAP).

## Discussion

The short median survival and poor prognosis of patients diagnosed with GB implicates a high need for novel therapeutic approaches. A recent review by Grek et al. highlighted the important role of Cx proteins in the microenvironment of malignant glioma [8]. Several studies have indicated Cx43, the most ubiquitous connexin, as an operator of tumor invasion, an indicator of tumor progression and an inducer of TMZ resistance in GB cells [8,19–24]. Cx43 peptidomimetics offer a therapeutic opportunity via mechanisms that are able to modulate gap junctional activity between tumor cells and cells in the peritumoral zone. Cx43 peptidomimetics can target Cx43-based GJs and/or hemichannels, as well as proteins that interact with Cx43 at the cytoplasmic site. Murphy et al. recently demonstrated that Cx43 is crucial for TMZ resistance and aCT1 (C-terminal mimetic peptide of Cx43), a selective inhibitor of Cx43 channels, could restore TMZ sensitivity in TMZ-resistant/Cx43-high GB cells [21]. aCT1 was also capable of inhibiting the growth of LN229/glioma stem cell tumors in mice treated with TMZ [25]. Moreover, combining aCT1 with TMZ further strengthened the inhibition of GB stem cell self-renewal and viability [25]. In addition to aCT1, a number of other Cx43 peptidomimetics including PEP-2, JM2, L2, Gap 26, Gap 27 and Gap 19 have currently been investigated for their therapeutic potential [8].

An alternative approach targeting gap junctional activity may involve the use of monoclonal antibodies against the second extracellular fragment of Cx43 (MAbE2Cx43) [26]. The efficiency of the antibodies was investigated using combinations of TMZ chemotherapy and fractionated radiotherapy in the C6 glioma rat model [27]. Treatment of GB with MAbE2Cx43 as a single agent showed significant inhibition of tumor growth. A combination of IV injection of MAbE2Cx43 and radiotherapy was demonstrated to be most effective for inhibition of tumor growth, possibly because of an increase in BBB permeability after irradiation. Treatment with chemotherapy and MAbE2Cx43 was not successful, probably caused by competitive inhibition between MAbE2Cx43 and TMZ [27].

Cx43-mediated GJC have been observed between tumor cells, between astrocytes and between tumor cells and astrocytes [8]. Chen et al. suggested tonabersat, a benzopyran derivate that binds to a unique stereoselective binding site in astrocytes, as a potential drug to treat established brain metastasis [12]. Tonabersat is an orally bioavailable gap junction modulator that inhibits the release of cytokines that protect brain metastatic cells against chemotherapeutic and physiological stress. The therapeutic effect of tonabersat (10 mg/kg per day, starting 14 days after cancer cell inoculation) as a single agent and in combination with carboplatin (5 mg/kg per 5 days, starting 14 days after cancer cell inoculation) has been investigated in mice using a brain metastatic model derived from mammary adenocarcinomas. Brain metastatic lesions were quantified based on bioluminescent imaging. Both treatment approaches significantly inhibited progression of the brain metastatic lesions [12].

In this preclinical study, we assessed the therapeutic potential of tonabersat in the F98 GB rat model. For this purpose, the geometric estimated mean tumor volumes were evaluated at several time points for four treatment groups (Control Group, ST Group, STCM Group and CM Group). The rats in the Control Group showed rapid GB proliferation, leading to early euthanasia due to clinical deterioration. GB proliferation was slowed down in the ST Group. Standard medical treatment with radiotherapy and TMZ chemotherapy significantly inhibited GB proliferation, confirming the effectiveness of standard medical treatment in the F98 GB rat model [17]. To assess the therapeutic potential of tonabersat, tonabersat was evaluated as a single agent and in combination with standard medical treatment. Our results demonstrated the efficacy of standard medical treatment supplemented with tonabersat as the geometric estimated mean tumor volumes were significantly lower in the STCM Group compared to the Control Group on day 10, 13 and 15. To evaluate if standard medical treatment supplemented with tonabersat was more effective then standard medical treatment, pairwise comparison of the estimated geometric mean tumor volumes between both groups was performed. Statistical analysis showed significant differences in estimated geometric mean tumor volumes from day 15 onwards. From day 20 onwards, the geometric mean tumor volume in the STCM Group is about 80% lower than the estimated geometric mean tumor volume in the ST Group. Our results indicate the adjuvant therapeutic potential of tonabersat for the treatment of GB when used in combination with radiotherapy and TMZ chemotherapy. This might reflect that tonabersat is active at the infiltrative border of the tumor, which can be confirmed by histological analysis that showed enhanced Cx43 expression and GFAP-positive reactive astrocytes at the peritumoral zone of GB, indicative for gliomagenesis, tumor progression and treatment resistance. Treatment of GB with tonabersat as a single agent was not successful as no significant reduction of GB growth was found. In contrast, Chen et al. demonstrated a significant reduced progression of brain metastatic lesions in mice when tonabersat was administrated as a single agent [12]. A possible explanation for this different result can be assigned to the complex and dual functions of connexins in cancer. Connexins can act as tumor suppressors or enhancers depending on the stage of carcinogenesis. Moreover, connexins are not only present in tumor tissue but also in host tissue [8,28–31].

**Fig 5:**
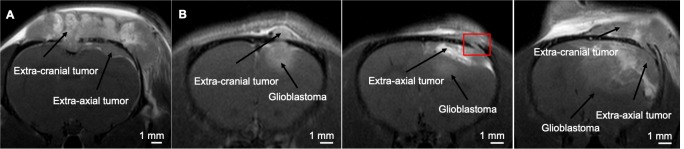
Extra-cranial and extra-axial tumor development. A. Extra-cranial and extra-axial tumor development on CE-T1w image. B. In vivo serial CE-T1w MRI scans in the same rat showing extra-axial tumor development. Invasion of cancer cells from the extra-cranial tumor through the cranial sutures leading to extra-axial tumor development indicated by the red box.

There are however a few shortcomings of our study. No survival analysis was performed because it is not ethically justified in animal experiments. Moreover, several rats (n = 6) needed to be euthanized because of extended extra-cranial and/or extra-axial tumor development (Fig 5). Extra-cranial tumor formation was probably due to backflow of tumor cells through the injection track leading to proliferation of the cancer cells on the skull. Radiotherapy only targeted intra-cerebral GB, allowing uncontrolled proliferation of the extra-cranial tumor followed by invasion through the cranial sutures and thus extra-axial tumor formation. These extra-axial tumors became large space‐occupying lesions necessitating euthanasia. Mainly the STCM Group developed extra-axial tumor growth, probably due to the extended lifespan as a result of the effective combination of RT, TMZ chemotherapy and Cx modulation with tonabersat. The uncontrolled proliferation of extra-cranial and extra-axial tumors allows us to assume that the effect of radiotherapy is of utmost importance in treatment of GB. Radiotherapy can increase the permeability of the blood brain barrier and therefore allow more efficient delivery of tonabersat to the tumor [32].

Another limitation of this study is related to the administration of radiotherapy using the SARRP. Rats receive radiotherapy in a single dose of 20 Gy instead of 30 fractions of 2 Gy. It is however stated that the biological effect of a single dose of 20 Gy should approximately equal the dose of 30 fractions of 2 Gy used in the clinic [17]. Nevertheless, differences in biological effect are still possible due to the higher dose.

In summary, our preclinical results using combinations of radiotherapy, chemotherapy and tonabersat provide a proof of concept for the adjuvant therapeutic potential of tonabersat for the treatment of GB.

## Supporting information

S1 Table Overview of cause of death for all rats. Rats were immediately euthanized when clinical and behavioral signs were observed.
(PDF)

S2 Table GB volumes. Tumor volumes were measured on CE-T1w MR images by manually outlining the hyperintense regions on individual slices.
(PDF)

S1 File The ARRIVE guidelines checklist. Completed ARRIVE Guidelines Checklist for reporting animal data in this manuscript.
(PDF)
