# Supplementary material for: Adjuvant therapeutic potential of tonabersat in the standard treatment of glioblastoma: A preclinical F98 glioblastoma rat model study
Source: PLoS One. 2019 Oct 21;14(10):e0224130. doi: 10.1371/journal.pone.0224130 (PMC6802836; doi:10.1371/journal.pone.0224130)
Supplement: S1 Table — Rats were immediately euthanized when clinical and behavioral signs were observed. (PDF) [file pone.0224130.s001.pdf]

	Day of euthanasia (*)	Cause of euthanasia (beeldvorming)
**Rat 1_Control**	Day 10	Pathological changes observed using brain MRI, weight loss, clinical signs caused by GB growth (balance problems)
**Rat 2_Control**	Day 13	Pathological changes observed using brain MRI, weight loss, clinical signs caused by GB growth (reduced activity)
**Rat 3_Control**	Day 13	Pathological changes observed using brain MRI, weight loss, clinical signs caused by GB growth (reduced activity)
**Rat 4_Control**	Day 13	Pathological changes observed using brain MRI, weight loss
**Rat 5_Control**	Day 8	Pathological changes observed using brain MRI, weight loss, clinical signs caused by GB growth (balance problems)
**Rat 6_Control**	Day 13	Pathological changes observed using brain MRI, weight loss, clinical signs caused by GB growth (balance problems)
**Rat 7_Control**	Day 15	Pathological changes observed using brain MRI, weight loss
**Rat 8_Control**	Day 15	Pathological changes observed using brain MRI, weight loss
**Rat 9_Control**	Day 15	Pathological changes observed using brain MRI, weight loss
**Rat 10_Control**	Day 8	Pathological changes observed using brain MRI, weight loss
**Rat 1_ST**	Day 19	Pathological changes observed using brain MRI, weight loss, clinical signs caused by GB growth (grooming)
**Rat 2_ST**	Day 21	Pathological changes observed using brain MRI, weight loss, clinical signs caused by GB growth (reduced activity)
**Rat 3_ST**	Day 33	Pathological changes observed using brain MRI, weight loss, clinical signs caused by GB growth (reduced activity)
**Rat 4_ST**	Day 25	Pathological changes observed using brain MRI, weight loss, clinical signs caused by GB growth (grooming, reduced activity)
**Rat 5_ST**	Day 31	Pathological changes observed using brain MRI, weight loss
**Rat 6_ST**	Day 39	Pathological changes observed using brain MRI, weight loss
**Rat 1_STCM**	Day 10	Balance problems, weight loss
**Rat 2_STCM**	Day 23	Pathological changes observed using brain MRI (extra-axial tumor growth), weight loss
**Rat 3_STCM**	Day 23	Pathological changes observed using brain MRI (extra-axial tumor growth), weight loss, clinical signs probably caused by extra-axial tumor growth
**Rat 4_STCM**	Day 37	Pathological changes observed using brain MRI (extra-axial tumor growth), weight loss
**Rat 5_STCM**	Day 37	Pathological changes observed using brain MRI (extra-axial tumor growth), weight loss
**Rat 6_STCM**	Day 35	Pathological changes observed using brain MRI, weight loss, clinical signs caused by GB growth (grooming, reduced activity)
**Rat 7_STCM**	Day 29	Pathological changes observed using brain MRI (extra-axial tumor growth), weight loss
**Rat 8_STCM**	Day 37	Pathological changes observed using brain MRI (extra-axial tumor growth), weight loss
**Rat 1_CM**	Day 17	Pathological changes observed using brain MRI, weight loss
**Rat 2_CM**	Day 10	Pathological changes observed using brain MRI, weight loss
**Rat 3_CM**	Day 10	Pathological changes observed using brain MRI, weight loss
**Rat 4_CM**	Day 10	Pathological changes observed using brain MRI, weight loss
**Rat 5_CM**	Day 13	Pathological changes observed using brain MRI, weight loss, clinical signs caused by GB growth (reduced activity)

(*) Day 1 = start of standard medical treatment or first control injection.
