# Supplementary material for: Adjuvant therapeutic potential of tonabersat in the standard treatment of glioblastoma: A preclinical F98 glioblastoma rat model study
Source: PLoS One. 2019 Oct 21;14(10):e0224130. doi: 10.1371/journal.pone.0224130 (PMC6802836; doi:10.1371/journal.pone.0224130)
Supplement: S2 Table — Tumor volumes were measured on CE-T1w MR images by manually outlining the hyperintense regions on individual slices. (PDF) [file pone.0224130.s002.pdf]

GB volume (mm^3)																		
	Confirmation	Day 3	Day 6	Day 10	Day 13	Day 15	Day 17	Day 19	Day 21	Day 23	Day 25	Day 27	Day 29	Day 31	Day 33	Day 35	Day 37	Day 39
**Rat 1_Control**	27,06	42,84	64,80	138,25														
**Rat 2_Control**	5,97	12,13	52,91	183,78	377,36													
**Rat 3_Control**	11,09	24,40	45,01	123,56	347,54													
**Rat 4_Control**	13,14	54,00	84,85	161,48	344,36													
**Rat 5_Control**	30,42	54,32	87,48															
**Rat 6_Control**	49,94	64,48	117,10	229,41	362,98													
**Rat 7_Control**	29,51	64,17	106,14	177,25	265,36	377,48												
**Rat 8_Control**	32,73	65,44	111,82	198,81	357,18	496,88												
**Rat 9_Control**	22,51	64,81	98,48	178,03	349,87	479,10												
**Rat 10_Control**	16,80	62,29	186,96															
**Rat 1_ST**	24,53	58,76	39,76	34,69	41,51	97,64	199,81	281,79										
**Rat 2_ST**	18,11	69,06	61,41	76,38	120,31	157,73	205,37	288,87	498,94									
**Rat 3_ST**	21,86	79,22	88,50	86,77	87,30	87,14	79,23	100,66	108,41	119,44	154,49	216,78	299,49	400,50	523,76			
**Rat 4_ST**	33,14	80,74	85,43	88,79	89,22	87,14	103,68	177,87	185,63	256,04	299,62							
**Rat 5_ST**	25,09	69,29	69,06	75,42	78,38	87,44	100,60	130,92	131,73	177,87	257,42	317,25	426,66	493,11				
**Rat 6_ST**	6,65	16,75	14,68	16,82	27,30	17,08	14,95	22,11	34,70	62,32	77,88	112,00	186,20	243,59	314,54	454,75	608,81	710,98
**Rat 1_STCM**	22,92	38,82	27,64	7,56														
**Rat 2_STCM**	16,88	32,01	42,93	41,52	50,25	55,59	50,45	51,02	47,94	46,41								
**Rat 3_STCM**	12,65	67,99	65,38	53,07	24,86	24,24	15,38	14,89	18,64	17,53								
**Rat 4_STCM**	24,00	47,52	47,03	33,05	35,84	31,26	36,87	34,25	31,03	52,55	64,46	80,92	117,21	173,84	183,98	255,85	392,97	
**Rat 5_STCM**	9,33	30,84	40,38	34,38	36,61	35,76	35,98	33,66	40,00	39,01	41,21	51,42	84,13	96,20	123,43	167,69	185,68	
**Rat 6_STCM**	12,95	36,38	34,96	46,60	42,97	33,50	32,44	34,81	33,30	47,32	62,28	82,53	135,32	201,29	224,67	305,55		
**Rat 7_STCM**	20,56	49,36	42,76	45,78	42,33	33,37	28,73	40,82	45,28	53,23	62,09	69,72	88,77					
**Rat 8_STCM**	11,50	20,40	19,23	18,83	19,80	19,75	20,20	17,43	16,83	19,36	17,41	28,71	31,06	40,50	82,55	83,24	118,79	
**Rat 1_CM**	24,02	51,83	102,24	176,72	240,27	365,52	385,93											
**Rat 2_CM**	44,65	97,33	185,53	258,25														
**Rat 3_CM**	21,72	58,19	128,18	254,39														
**Rat 4_CM**	50,44	84,82	170,62	319,04														
**Rat 5_CM**	55,09	91,89	130,89	259,31	340,63													
